# Synopsis of the tribe Stipeae (Poaceae) in Nepal

**DOI:** 10.3897/phytokeys.128.34637

**Published:** 2019-08-19

**Authors:** Marcin Nobis, Polina D. Gudkova, Colin A. Pendry

**Affiliations:** 1 Institute of Botany, Faculty of Biology, Jagiellonian University, Gronostajowa 3 st., 30-387 Kraków, Poland Jagiellonian University Kraków Poland; 2 Research laboratory “Herbarium”, National Research Tomsk State University, 36 Lenin ave., 634050, Tomsk, Russia National Research Tomsk State University Tomsk Russia; 3 Faculty of Biology, Altai State University, 61 Lenin ave., 6560496, Barnaul, Russia Altai State University Barnaul Russia; 4 Royal Botanic Garden Edinburgh, 20a Inverleith Row, Edinburgh EH3 5LR, Scotland, UK Royal Botanic Garden Edinburgh Edinburgh United Kingdom

**Keywords:** Checklist, Identification key, Nepal, Poaceae, Stipeae

## Abstract

In Nepal the Stipeae consists of six genera: *Achnatherum*, *Orthoraphium*, *Piptatherum*, *Ptilagrostis*, *Stipa*, *Trikeraia*, and 15 species. Two new combinations, *Ptilagrostis duthiei* (Hook. f.) M.Nobis & P.D.Gudkova and *Achnatherum staintonii* (Bor) M.Nobis & P.D.Gudkova, are proposed, and new country records for *Stipa klimesii*, *Ptilagrostis dichotoma*, *Ptilagrostis concinna* and *Achnatherum jacquemontii* are reported. The records of *Stipa roborowskyi*, *S. przewalskyi*, *S. capillata*, *S. consanguinea*, *S. mongholica*, and *S. sibirica*, previously thought to occur in Nepal were based on misidentifications, and these have been excluded from the list of Nepalese Stipeae. We present keys for the identification of genera and species, and a checklist including information on nomenclatural types, regional and national distribution, and habitat. A lectotype is designated for *Stipa brandisii* Mez.

## Introduction

The tribe Stipeae L. (feather grasses) is composed of extratropical and high-mountain grasses consisting of about 680 species distributed on all continents except Antarctica (Tzvelev 1977; Barkworth 2007; Romaschenko et al. 2008, 2010, 2011, 2012; Soreng et al. 2003, 2015, 2017, Nobis et al. 2019). It is an early divergent, highly specialized, monophyletic lineage within the subfamily Pooideae Benth. The Stipeae are characterized by their single-flowered spikelets without rachilla extensions, lemmas with apical awns where the awn is the result of fusion between the central and two lateral vascular traces, florets with three (rarely two) lodicules, and usually the palea is concealed by the lemma (if the palea is exposed when the floret is closed, then the palea is coriaceous (Roshevitz 1934; Tzvelev 1976; Freitag1985).

Although agrostologists have maintained a broad concept of the genus *Stipa* L. since its description (Hitchcock 1935, Clayton and Renvoize 1986; Freitag 1985; Columbus et al. 2019 and others), recent molecular phylogenetic studies suggest that ca. 34 genera should be recognized within the tribe (Hamasha et al. 2012; Nobis et al. 2019; Peterson et al. 2019). However, the species composition of some genera still requires further study.

All previous treatments of the Stipeae in Nepal have followed a broad generic concept. The Annotated checklist of the flowering plants of Nepal (Press et al. 2000, http://www.efloras.org/flora_page.aspx?flora_id=110) and the Flora of Mustang, Nepal (Ohba et al. 2008) recognized 11 species of *Stipa*: *Stipa breviflora* Griseb., *S. capillata* L., *S. consanguinea* Trin. & Rupr., *S. duthiei* Hook. f., *S. koelzii* R.R.Stewart, *S. mongholica* Turcz. ex Trin., *S. przewalskyi* Roshev., *S. roborowskyi* Roshev., *S. roylei* (Nees) Mez., *S. sibirica* (L.) Lam. and *S. staintonii* Bor and three species of *Oryzopsis*: *O. gracilis* (Mez) Pilg., *O. lateralis* (Regel) Stapf ex Hook. f. and- *O. munroi* Stapf ex Hook.f. Two other species have recently been reported from Nepal, *Ptilagrostis milleri* (Noltie) M.Nobis & A.Nobis (=*S. milleri* Noltie), and *S. krylovii* Roshev. (Nobis et al. 2015; Gudkova et al. 2017a). Unfortunately, identification of Nepalese feather grasses is difficult due to the lack of recent, comprehensive, regional taxonomic studies. The main goal of this paper is to provide an identification key and checklist including information on types, nomenclature, distribution, and habitat for all Nepalese species of Stipeae.

## Materials and methods

**Figure 1.**
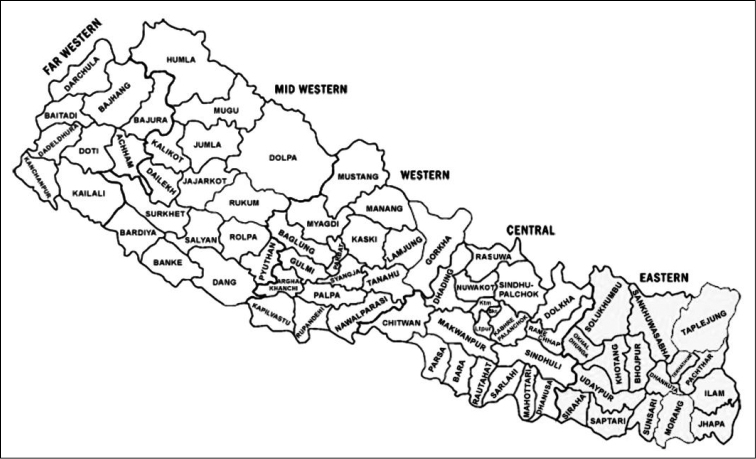
Districts of Nepal.

Our treatment is based on herbarium specimens deposited in BM, E, GOET, K, KATH, KRA, KUN, LE, M, NY, P (Thiers 2018). Each species is listed with complete nomenclatural and type information (the type specimens examined has exclamation mark after a herbarium code) synonyms, habitat requirements, and Nepalese and general distribution. The distribution within Nepal is given by District (Fig. 1). Elevation ranges and habitat requirements have been determined from herbarium specimen labels and from the literature.

### Morphological analyses

Nineteen morphological characters scored for each taxon were included in the analysis (Table 1). Each species was treated as an Operational Taxonomic Unit (OTU) following Sokal and Sneath (1963). Cluster analysis was performed on all OTUs to estimate morphological similarities among the species. The similarities among OTUs were calculated using Gower’s general similarity coefficient. A cluster analysis (UPGMA) was carried out using PAST software (Hammer et al. 2001).

**Table 1.** Morphological characters and character states.

Characters	States
**Macromorphological characters**:	
Length of anthecium (lemma + callus) [mm]	mean length
Length of callus [mm]	mean length
Ratio lemma / palea	subequal (1); lemma longer than palea (2)
No. of awn geniculations	without geniculations (0); unigeniculate (1); bigeniculate (2)
Length of awn [mm]	mean length
Hairs on column [mm]	mean length
Hairs on seta [mm]	mean length
Length of glumes [mm]	mean length
Apex of glumes	twisted (1), straight (2)
Ligules of vegetative leaves [mm]	mean length
Ratio lower glume / upper glume	subequal (1); lower longer than upper (2)
Hard prickles at lemma apex	absent (1); present (2)
**Micromorphological characters of the lemma epidermis**:	
Length of long cells	1–3(–5) times as long as wide (1); (4–)5–9(–11) times as long as wide (2), as wide as long (3)
Side walls of long cells	not thickened (1), thickened (2)
Presence of hooks	frequent (more than 12 on area of 0.015 mm^2^) (1); sparse (less than 12 on area of 0.015 mm^2^) (2), absent (3)
Presence of silica cells	frequent (more than 20 per area of 0.015 mm^2^) (1); sparse (less than 20 per area of 0.015 mm^2^) (2); rare (less than 5 per area of 0.015 mm^2^) (3)
Constriction of silica cells	with constrictions (1), without constrictions (2)
Shape of silica cells	ovate (1); elongated to ovate (2), elliptic or reniform (3)

### Stipeae Dumort., Observ. Gramin. Belg. 83, 88, 134 (1824)

Plants perennial, usually cespitose, occasionally rhizomatous. Culms erect, unbranched. Leaf blades flat or convolute, abaxial surface smooth, scabrous or pubescent, adaxial surface prominently ribbed, with 0.05–1 mm long hairs. Ligules membranous. Inflorescence a dense or open panicle. Spikelets with one bisexual floret. Glumes clearly unequal to subequal, membranous, obtuse or acute, tapering into a long tip. Awns scabrid to plumose, straight, uni- or bi-geniculate. Lemmas narrowly lanceolate, terete, usually leathery, usually hairy. Callus rounded or acute to sharply pointed.

## Results and discussion

**Figure 2.**
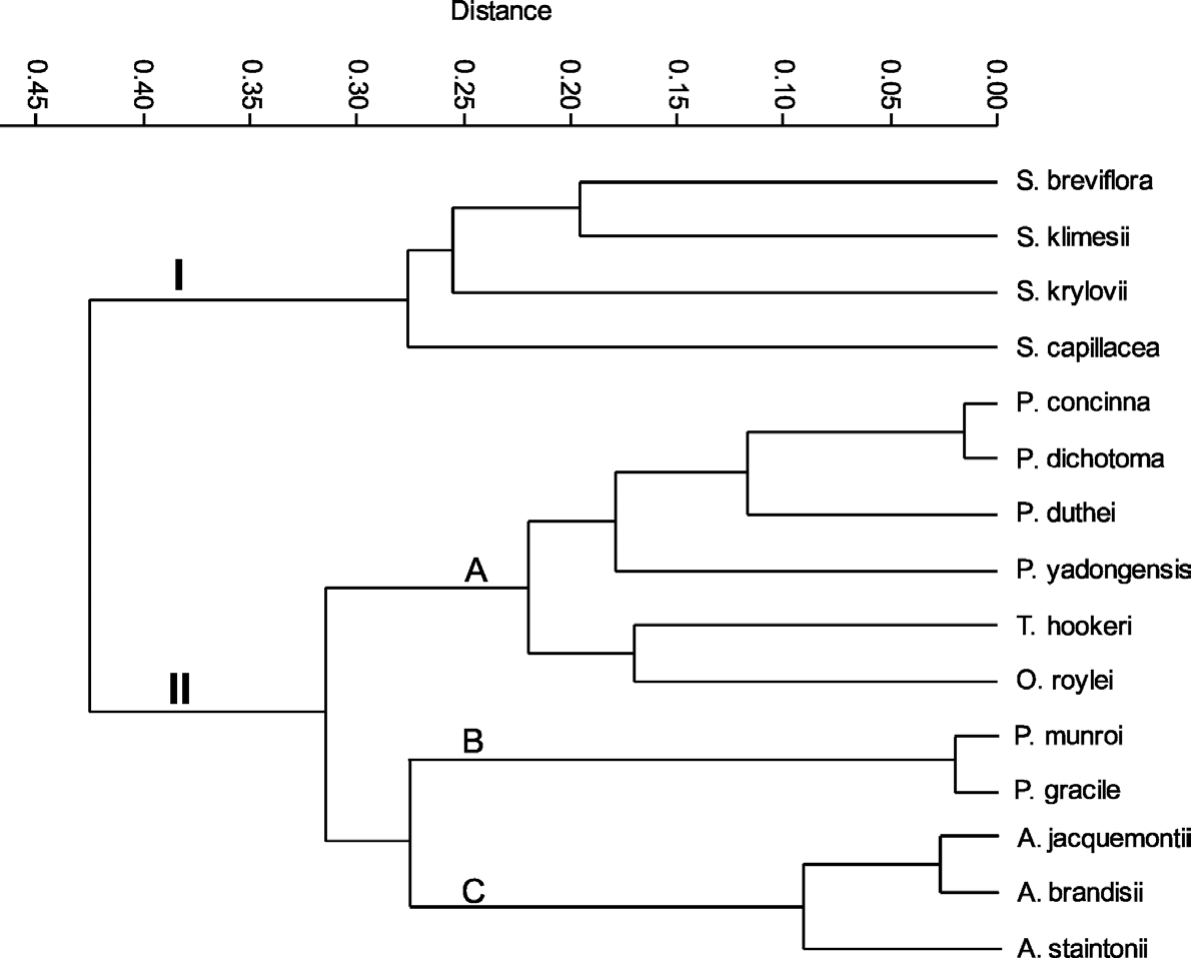
Cluster analysis (UPGMA method of classification and Gower’s general similarity coefficient) performed on 16 qualitative morphological characters for all of the Nepalese members from the tribe Stipeae.

**Figure 3.**
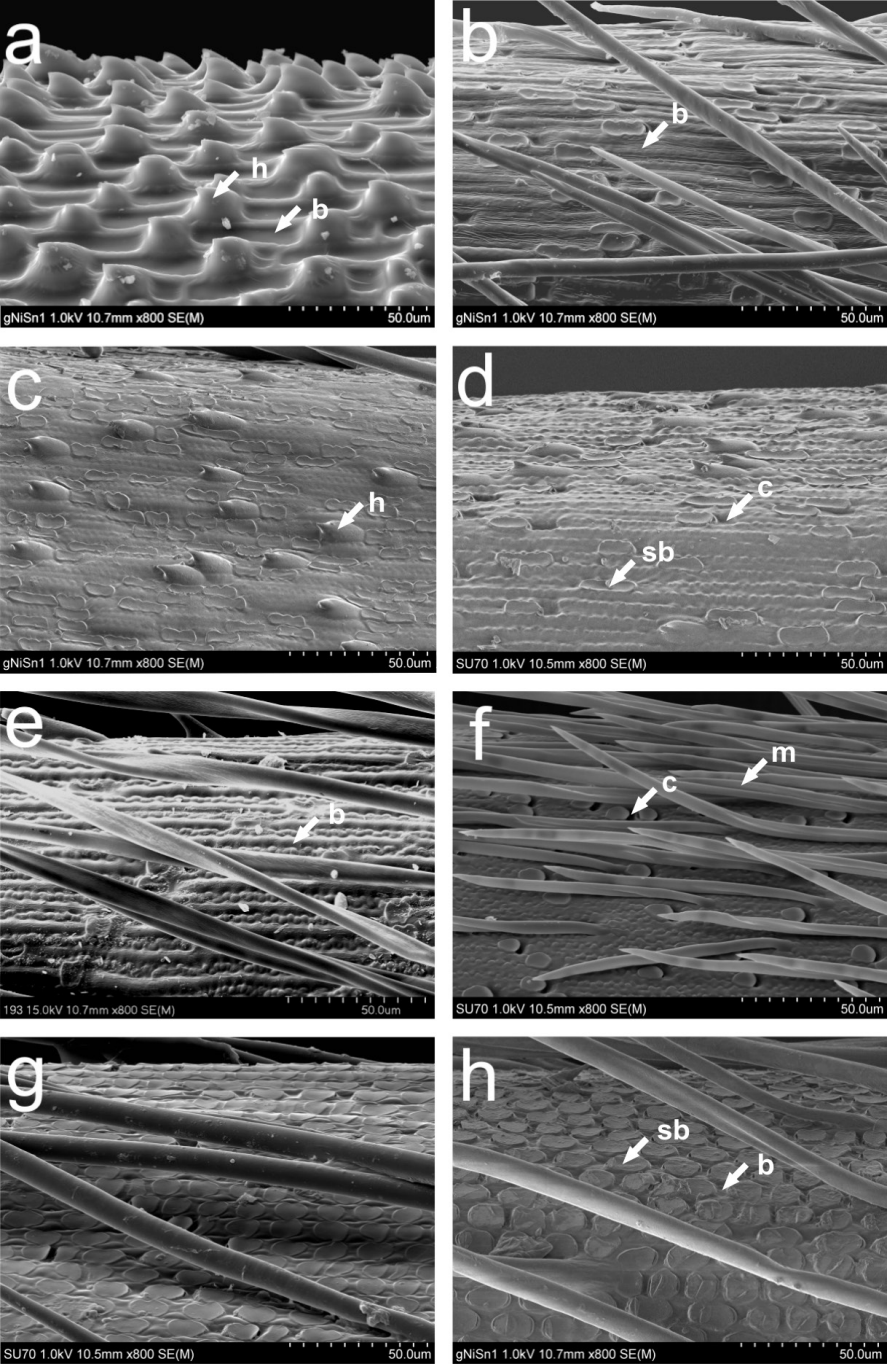
Lemma epidermal patterns (LEPs) of Old World Stipeae: **a***Stipa breviflora* [Kyrgyzstan, near Issyk-Kul Lake, *M. Nobis* (KRA)] **b***Ptilagrostis concinna* [India, Ladakh, Himalayas, *L. Klimeš* (KRA)] **c***Ptilagrostis duthiei* [India, Himalayas, *J.F. Duthie 3585* (LE) **d***Orthoraphium roylei* [India, Himalayas, *J.F. Duthie 3568* (LE)] **e***Trikeraia hookeri* [China, Tibet (PE 718306)] **f***Piptatherum munroi* [Nepal, Solukhumbu, *M.F. Watson et al. DNEP3 AX33* (E)] **g***Achnatherum brandisii* [India, Kashmir, *R.R. Stewart 18120* (NY)] **h***Achnatherum staintonii* [Nepal, Mustang, *M.A. Farille 81-340* (E)]. Annotations: b – basal cells, c – cork cells, sb – silica bodies, m – macro-hairs.

Detailed analyses of macro- and micromorphological structures of the lemma epidermis of Nepalese species of Stipeae confirmed that they form two main clusters, one with three subclusters (Fig. 2). The clusters correspond to the four lemma epidermal patterns (LEP): *Stipa*-like, *Ptilagrostis*-like, *Piptatherum*-like and *Achnatherum*-like (Fig. 3). The taxa from cluster I belonging to *Stipa* have long cells and hooks on the lemma epidermis in an ordered saw-like pattern (Romaschenko et al. 2012; Fig. 3a). Within subcluster A of cluster II, there are three genera, *Ptilagrostis*, *Trikeraia* and *Orthoraphium* (Fig. 2), that have LEPs dominated by elongated basal cells, frequent silica bodies and cork cells (Fig. 3b–d, h). However, the presence of deflexed, hard prickles in the case of *Orthoraphium roylei* (Fig. 3d), as well as 2–3 mm long awn-like lemma lobes in the case of *Trikeraia hookeri* (Fig. 3e) are unique characters which distinguish them from other members of the subcluster. Subcluster B comprises species from the genus *Piptatherum*. These species differ from those in subcluster A in their extremely short callus, less numerous and rounded silica bodies on the lemma surface (Fig. 3f). Taxa in subcluster C, all of which belong to the genus *Achnatherum*, have a maize-like type of LEP (Romaschenko et al. 2012), characterized by numerous silica bodies and very short basal cells (Fig. 3g–h). All of these species have lemmas distinctly longer than paleas.

### Key to genera

**Table:** 

1	Lemma with deflexed (retrorse), apical prickles	*** Orthoraphium ***
–	Lemma lacking deflexed, apical prickles	**2**
2	Lemma lobes awn-like, 2–3 mm long, setaceous	*** Trikeraia ***
–	Lemma without awn-like lobes, lobes (if present) flat and less than 1 mm long	**3**
3	Awns straight, scabrous. Anthecium usually dorsally compressed. Callus up to 0.3 mm long	*** Piptatherum ***
–	Awns geniculate, scabrous or variously pilose. Anthecium not compressed or laterally compressed. Callus longer than 0.3 mm	**4**
4	Callus longer than 0.9 mm. Lemma epidermis with numerous minute hooks (visible under high magnification)	*** Stipa ***
–	Callus up to 0.8 mm long. Lemma epidermis smooth or rarely with infrequent minute hooks	**5**
5	Lower segment of awn pilose, with hairs over 0.3 mm long. Surface of lemma epidermis covered with elongated basal cells (4–11 times longer than wider) and occasional, 1–3-constricted silica bodies	*** Ptilagrostis ***
–	Lower segment of awn scabrous, with hairs up to 0.1 mm long. Surface of lemma epidermis covered with rounded or once-constricted silica, underlying cells as wide as long or wider than longer	*** Achnatherum ***

#### Orthoraphium

Taxon classificationPlantaePoalesPoaceae

Nees, Proc. Linn. Soc. Lond. 1: 94 (1841)

388F4DC2FEB2501192FB44591B750571

##### Type.

*Orthoraphium roylei* Nees.

#### Orthoraphium roylei

Taxon classificationPlantaePoalesPoaceae

Nees, Proc. Linn. Soc. Lond. 1: 94 (1841).

3277C9C8961C522EACA046C374388E20

≡ Stipa orthoraphium Steudel, Syn. Pl. Glumac. 1: 131 (1855) nom. superfl.;
≡ Stipa roylei (Nees) Duthie, Grasses North-Western India 27 (1883);
≡ Stipa roylei (Nees) Mez, Repert. Spec. Nov. Regni Veg. 17: 207 (1921).

##### Type.

(India, W Himalaya) Kadarkanal, *Royle* (holotype: LIV).

##### General distribution.

Himalayas: Bhutan, S China, Ladakh, N Myanmar, N India, Nepal (Bor 1960; Freitag 1985; Wu and Phillips 2006).

##### Distribution in Nepal.

Baglung, Bajura, Darchula, Dolkha, Humla, Jumla, Lalitpur, Mugu, Myagdi, Ramechhap, Rasuwa, Rukum, Sankhuwasabha, Solukhumbu, Taplejung.

##### Habitat.

Alpine meadows, *Rhododendron* scrub, oak-laurel forests.

##### Altitudinal range.

2200–4000 m.

##### Selected Nepalese specimens studied.

**Baglung**: Dhorpatan, Vallee de Dhorpatan du cote est, 28°29'18"N, 83°4'1"E, 2800–3350 m, 16 Aug. 1981, *M. Farille 81-168* (E). **Bajura**: Birseni – Porakya, 2250 m, 12 Aug. 1991, *K.R. Rajbhandari 14829* (KATH). **Darchula**: Dopakhe, Dandar – Dopakhe, rocky slope, 2200 m, 28 Aug. 1980, *K.R. Rajbhandari & K.J. Malla 5684* (KATH). **Dolkha**: Gyalche Kharka – Thang Dingma, 3100 m, 1 Sep. 1983, *K.R. Rajbhandari 9744* (KATH); Bhitte Kharka – Patlo Pokhari, shady place in forest, 3700 m, 12 Sep. 1983, *K.R. Rajbhandari 10123* (KATH); Bhitte Kharka – Patlo Pokhari, 3800 m, 12 Sep. 1983, *K.R. Rajbhandari 10164* (KATH). **Humla**: Tambe Danda, 2750 m, Q. semecarpifolia forest, 15 Aug. 1977, *K.R. Rajbhandari & B. Roy 2268* (KATH). **Jumla**: Jumla, 29°16'31"N, 82°11'0"E, *H. Tabata, K.R. Rajbhandari & K. Tsuchiya 9327* (KATH). **Lalitpur**: Phulchoki, 27°34'14"N, 85°24'4"E, 2600 m, 14 Oct. 1990, *K.R. Rajbhandari 4122* (KATH). **Mugu**: Chankheli Lagna, 29°38'21"N, 82°6'52"E, 3500 m, 9 Aug. 1979, *K.R. Rajbhandari & B. Roy 4445* (KATH). **Myagdi**: Gurjakhani, North-West of Gurjakhani, 28°36'N, 83°13'E, 3480–3490 m, 31 Jul. 1954, *J.D.A. Stainton, W.R. Sykes & L.H.J. Williams 3685* (BM, E, K). **Ramechhap**: Khola Kharka – Thare, 3600 m, 22 Jul. 1985, *H. Ohba et al. 60583* (KATH); Bhandar – Deorali – Khasrubus – Shivalaya, 2600 m, *H. Ohba et al. 62276* (KATH). **Rasuwa**: Rupchet Kharka - Balchagam, 3200 m, 16 Aug. 1994, *K.R. Rajbhandari 17910* (KATH); Laurebina Yak, 28°5'32"N, 85°22'52"E, 3450 m, 30 Jul. 1995, *T. Hoshino et al. 9537289* (KATH). **Rukum**: Sing Khola – Farkama 3636 m, 18 Sep. 1976, *H. Tabata, K.R. Rajbhandari & K. Tsuchiya 3720* (KATH). **Sankhuwasabha**: Hile Ghot, 27°24'N, 87°26'E, 3500 m, 20 Aug. 1972, *J.F. Dobremez 1594* (BM, E). **Solu Khumbu**: Imja Khola Valley, Omoga, sandy clay, floor of north-west/south-east river valley, west facing slope, mossy slope in shade, 27°50'38"N, 86°47'10"E, 3600–2300 m, 26 Sep. 2005, DNEP3 AX131 (E, KATH); Chaurikharka, Q.semecarpifolia forest, mossy slope, 27°41'46"N, 86°43'31"E, 2729 m, 30 Sep. 2005, *DNEP3 BY229* (E, KATH); Dudh Kund – Samakang, 27°41'N, 86°50'E, 3500 m, 24 Aug. 1995, *F. Miyamoto et al. 9592410* (E); Samakang Kharka 27°41'N, 86°50'E, 3500 m, 24 Aug. 1995, *F. Miyamoto et al. 9592410* (KATH); Beni Kharka, 3100 m, 3 Sep. 1985, *H. Ohba et al. 62035* (KATH); Beni Kharka, 3600–2300 m, 2 Sep. 1985, *H. Ohba et al. 61907* (KATH); Loding, 27°32'N, 86°32'E, 2700 m, 5 Sep. 1985, *H. Ohba et al. 62108* (KATH); *62155*; Pike Bhanjyang, 3700 m, 6 Sep. 1985, *H. Ohba et al. 62155* (KATH); Pike Dongshar, Rhododendron campanulatum thicket, 27°30'N, 86°27'E, 2300–3600 m, 9 Sep. 1985, *H. Ohba et al. 62192* (KATH); Rangdu Kharka, 27°8'N, 86°48'E 3550 m, 9 Aug. 1997, *K.R. Rajbhandari 9740203* (KATH); Tangnag – Mosom Kharka, in forest on mossy ground, 3700 m, 21 Aug. 1997, *K.R. Rajbhandari 9740472* (KATH); Beni – Tokchardingma, Basa Valley, 27°32'59"N, 86°35'13"E, 3750 m, 2 Aug. 1995, *K. Tsuchiya 40841* (KATH); Sengephuk, Beni VDC, 3580 m, 29 Aug. 1995, *K. Tsuchiya 41782* (KATH); Luminasa, Basa Valley, Beni, 3780 m, 8 Aug. 1995, *K. Tsuchiya* 42188 (KATH). **Taplejung**: forest ridge et Manedhanjang, N end of Milke Danda Ridge, Rhododendron / Bamboo forest, caespitose grass, 27°27'N, 87°28'E, 3340 m, 22 Oct. 1991, *D.G. Long et al. 966* (E, KATH); Topke Gola, Arun-Tamur watershed S of Topke Gola, on slopes, 27°38'25"N, 87°34'59"E, 3940 m, 13 Sep. 1956, *J.D.A. Stainton 1728* (BM, E).

#### Stipa

Taxon classificationPlantaePoalesPoaceae

L., Sp. Pl. 1: 78 (1753)

804F802A946C563C9139BE8965771259

##### Type.

*Stipa pennata* L.

### Key to the genus *Stipa*

**Table:** 

1	Upper part of awn (seta) scabrous, with hairs up to 0.4 mm long	**2**
–	Upper part of awn (seta) pilose, with hairs over 0.4 mm long	**3**
2	Upper part of awn and tips of glumes spirally twisted	***S. capillacea***
–	Upper part of awn and tips of glumes not twisted	***S. krylovii***
3	Ligules of vegetative shoots up to 0.3 mm long. Awn column with 0.6–0.7 mm long hairs	***S. breviflora***
–	Ligules of vegetative shoots over 2 mm long. Awn column with 1.5–2.5 mm long hairs	***S. klimesii***

#### Stipa breviflora

Taxon classificationPlantaePoalesPoaceae

Griseb., Nachr. Ges. Wiss. Göttingen, Math.-Phys. Kl. 3:82 (1868).

8A8EE1A845E35A58B2ADDF8B36ED47FC

= S. aliciae Kanitz, Növényt. Gyujtesek Eredm. Grof Szechenyi Bela Keletazsiai Utjabol 61, t. 7 (1891).

##### Type.

(China) Tibet, Gnari (Nari) Khorsum, *Schlagentweit 7105* (holotype GOET!, isotype LE!).

##### General distribution.

China, Kyrgyzstan, N India, Mongolia, Nepal (Grubov 1982; Tzvelev 1968, 1976; Freitag 1985; Wu and Phillips 2006).

##### Distribution in Nepal.

Mustang.

##### Habitat.

High altitude steppes, scree.

##### Altitudinal range.

2750–3600 m.

##### Selected specimens studied.

**Mustang**: Entre Jomsom et Kagbheni, dans la steppe aride a *Caragana gerardiana* et *C. brevispina* (limite), 28°46'51"N, 83°43'27"E, 2750 m, 17 Sep. 1981, *M.A. Farille 81-362* (E); Muktinath, on open slopes near cultivations, 28°48'58"N, 83°51'47"E, 3640 m, 8 Jun 1954, *J.D.A. Stainton, W.R. Sykes & L.H.J Williams 5647* (E, K).

#### Stipa klimesii

Taxon classificationPlantaePoalesPoaceae

M.Nobis, Phytotaxa 174(3): 166–168 [174–176] (2014).

B5D3632F036D53DB88547F8386323F45

= Stipa basiplumosa Munro ex Hook. f., Fl. Brit. India 7(22): 229 (1896) var. longearistata Munro ex Hook. f., Fl. Brit. India 7(22): 229 (1896).

##### Type.

India, NW India, Jammu and Kashmir State, Ladakh, Indus Vy: Zhung (Leh), Ganglas – upper part, springs with drinking water, 3880–4000 m, 30 Jul. 2001, 34°12.3'N, 77°36.8'E, *L. Klimeš 1155, 1156* (holotype KRA!, isotype PRA!).

##### General distribution.

Bhutan, China (Tibet), India (Ladakh, Sikkim), Nepal, Pakistan, (Nobis et al. 2014, 2015).

##### Distribution in Nepal.

Mustang.

##### Habitat.

High mountain steppes and alpine mats, among subalpine shrubs and on rocky ledges.

##### Altitudinal range.

3500–5000 m.

##### Notes.

These specimens were previously identified as *S. roborowskyi*, but this species does not occur in Nepal. This species differs from *S. klimesii* in having shorter ligules on the vegetative shoots [0.5–1.5(–2) vs. (2–)3.5–7.5(–9) mm], shorter anthecium [(6–)6.5–7.5(–7.7) vs. (7–)8.3–9.5(–10) mm] and shorter hairs on seta [(0.3)0.5–1.1(–1.4) vs. (1–)1.3–2(–2.3) mm long, respectively].

##### Selected specimens studied.

**Mustang**: Damodar, on dry sandy slope, 29°11'N, 83°58'E, 28 Jul. 1979, *P.R. Shakya, S.R. Adhikari & K.R. Amatya 5111* (KATH); Kunda, 28°59'9"N, 84°9'26"E, 4720–4740 m, 11 Aug. 2001, *S. Noshiro, M. Amano & T. Kurosawa 20104179* (KATH).

#### Stipa krylovii

Taxon classificationPlantaePoalesPoaceae

Roshev., Izv. Glavn. Bot. Sada S.S.S.R. 28: 379 (1929).

41A6183EF4ED55F98282701FCECF19DC

≡ S. sareptana subsp. krylovii (Roshev.) Cui, Fl. Xinjiang. 6: 299 (1996);
≡ S. sareptana var. krylovii (Roshev.) Kuo & Sun, Fl. Reipubl. Popularis Sin. 9(3): 275, pl. 65, f. 37–41 (1987).
= S. capillata var. coronata Roshev., Fl. Aziat. Ross. 1(12): 168, pl. 8, 8b (1916);
= S. densiflora P.A.Smirn., Repert. Spec. Nov. Regni Veg. 26: 265 (1929) hom. illeg. non Hughes;
= S. densa P.A.Smirn., Del. sem. Hort. Bot. Univ. Mosquensis 15 (1930);
= S. decipiens P.A.Smirn., Ucen. Zap. Moskovsk. Gosud. Univ. 2: 338 (1934).

##### Type.

Selenginskaya Dauriya, gory mezhdu Temnikom i Dzhidoi, yugo-zapadnaya chast khr. Borgoiskogo, na sklonakh so stepnoi rastitelnostyu, 28 Jul. 1912, *V. Smirnov 524* (lectotype: LE!, designated by Tzvelev 1976).

##### General distribution.

Widely distributed throughout Central Asia (Eastern Kazakhstan, Russia (Siberia: Altai, Khakasiya, Tuva, South Krasnoyarsk, Irkutsk, Buryatiya, Chita, South Yakutia); China (Gansu, Hebei, Nei Mongol, Ningxia, Qinghai, Shanxi, Xinjiang, Xizang), Mongolia, eastern Kyrgyzstan, Tajikistan (Pamir), North India, Nepal. (Tzvelev 1968, 1976; Freitag 1985; Wu and Phillips 2006; Gudkova et al. 2017a, 2017b).

##### Distribution in Nepal.

Mustang (Gudkova et al. 2017a).

##### Habitat.

High mountain semi-desert.

##### Altitude range.

3900–4000 m.

##### Notes.

These specimens were previously identified as *S. capillata*, but this species does not occur in Nepal. *Stipa krylovii* differs from *S. capillata* mainly in having a ring of hairs at the top of the lemma.

##### Selected specimens studied.

**Mustang**: s.loc., on dry sandy ground, 29°14'N, 83°52'E, 13000 ft, 3 Aug. 1954, *Stainton*, *Sykes*, *Williams 2161* (E, K, BM).

#### Stipa capillacea

Taxon classificationPlantaePoalesPoaceae

Keng, Sunyatsenia 6(2): 100, pl. 15 (1941).

C9490C7C9EA55DAD993ABCAA85FD7536

= S. koelzii R.R.Stewart, Brittonia 5: 441 (1945).

##### Type.

Open grass land in rear of Shaowusze Agricultural Station, Taining district, Sikang province, 22 Jul. 1940, *K.L. Chü 7449* (holotype: N, isotype: PE!).

##### General distribution.

Bhutan, S China, N India, Nepal, Pakistan, (Freitag 1985; Noltie 2000; Wu and Phillips 2006).

##### Distribution in Nepal.

Mustang, Rasuwa, Solukhumbu.

##### Habitat.

Alpine meadows.

##### Altitudinal range.

2800–4100 m.

##### Note.

These specimens were previously identified as *Stipa consanguinea*, but this species does not occur in Nepal. *Stipa capillacea* differs from other species of the genus in awns twisted together at top of panicle.

##### Selected specimens studied.

**Mustang**: Kali Gandaki, Thulo Bugin, ESE Lete, S- facing steep slope, 28°38'4"N, 83°36'20"E, 2820 m, 10 Oct. 1977, *G. Miehe 762b* (BM); at edge of field, 13000 ft, 3 Aug. 1954, *J.D.A. Stainton, W.R. Sykes & L.H.J. Williams 2150* (BM, K); **Rasuwa**: Langtang, 28°13'N, 85°3'E, 3900 m, 7 Aug. 1970 *J.F. Dobremez 522* (BM, E); Kyanjin Gompa, 28°12'42"N, 85°34'1"E 11500–12500 ft, 10 Aug. 1969, *A. Richard 103* (BM); Ganesh Himal, 28°20'N, 85°10'E, Apr. 1975 - May 1975, *B. Yon 252* (E). **Solukhumbu**: Orsho, east facing slope open ground, grazed area with scattered Juniperus indica, 27°52'15"N, 86°48'44"E, 4100 m, 19 Sep. 2005, *DNEP3 AX83* (E, KATH).

#### Ptilagrostis

Taxon classificationPlantaePoalesPoaceae

Griseb., Fl. Ross. 4(13): 447 (1852)

CF9DB8F5E4385D8BB2E6461981EB4764

##### Type.

*Ptilagrostis mongholica* (Turcz. ex Trin.) Griseb.

### Key to the genus *Ptilagrostis*

**Table:** 

1	Awn with 0.3–0.5 mm long hairs on column. Seta scabrous	***P. duthiei***
–	Awn variously pilose on column, with hairs over 1 mm long. Seta with 0.5–1.5 mm long hairs	**2**
2	Glumes, lemma and palea distinctly unequal (lower glume 1.5–3.5 mm longer than the upper and lemma 1–2.5 mm longer than palea)	***P. yadongensis***
–	Glumes, lemma and palea equal or only slightly unequal	**3**
3	Panicles open, 3–5 cm wide, branches up to 6 cm long, spreading	***P. dichotoma***
–	Panicles compressed, 0.7–2 cm wide, branches 0.3–2.8 cm long, suberect or narrowly ascending	***P. concinna***

#### Ptilagrostis duthiei

Taxon classificationPlantaePoalesPoaceae

(Hook.f.) M.Nobis & P.D.Gudkova comb. nov.

1BE31E1DD7FE5BC982CE9B0927828736

urn:lsid:ipni.org:names:77200949-1

Stipa duthiei Hook.f., Fl. Brit. India 7: 232, 1896. (Basionym).
≡ Achnatherum duthiei (Hook.f.) Kuo & Lu, Fl. Reipubl. Popularis Sin. 9(3): 322, pl. 80, f. 9–14 (1987).

##### Type.

[India] Tehri Garwhal, Lekhus, below Srikanta, 12000–13000ft, 11 Aug. 1853, *Duthie 273* (holotype K 32097!).

##### General distribution.

China, N India, Kashmir, Nepal (Freitag 1985; Wu and Phillips 2006).

##### Distribution in Nepal.

Myagdi.

##### Habitat.

Mountain shrublands.

##### Altitudinal range.

3400–3800 m.

##### Selected specimens studied.

**Myagdi**: North of Barse, among dwarf Rhododendron, 3940 m, [28°35'N, 83°11'E], 14 Aug. 1954, *J.D.A. Stainton, W.R. Sykes & L.H.J. Williams 3844* (E 690624).

#### Ptilagrostis dichotoma

Taxon classificationPlantaePoalesPoaceae

Keng ex Tzvelev, Rast. Tsentr. Azii 4: 43 (1968).

518A082CB22A55AF8CAFBBF7DE61A68B

##### Type.

China, Kansu and Tsinghai border [in regione opp. Labrang], *I.C. Wu 478* (holotype N, isotype LE!).

##### General distribution.

Bhutan, Birma, China (Tibet), N India, Nepal (Wu and Phillips 2006; Nobis et al. unpbl).

##### Distribution in Nepal.

Bajhang, Bajura, Dolakha, Jumla, Mustang, Myagdi, Ramechhap, Rasuwa, Rukum, Solukhumbu.

##### Habitat.

Alpine meadows, grassy mountain slopes.

##### Altitudinal range.

3300–5000 m.

##### Note.

These specimens were previously identified as *Ptilagrostis mongholica* [=*Stipa mongholica*] (Bor 1960; Freitag 1985), but the two species are easily distinguished as *P. dichotoma* has a tuft of short hairs at the apex of anthers (glabrous in *P. mongholica*). They are disjunctly distributed with *P. dichotoma* found in the mountains of southern-central Asia while *P. mongholica* occurs mainly in the mountains of northern-central Asia; (Tzvelev 1968; Wu and Phillips 2006).

##### Selected specimens studied.

**Bajhang**: Manane Lekh 29°36'45"N, 80°59'35"E, 3830 m, 14 Jul. 2009, Bajhang 09 20917078 (E, KATH); Saipal, 29°57'51"N, 81°13'6"E, 3909 m, *H. Tabata, K.R. Rajbhandari & K. Tsuchiya 1808* (KATH); Saipal, 29°57'51"N, 81°13'6"E, 30 Jul. 1976, *H. Tabata, K.R. Rajbhandari & K. Tsuchiya 4024* (KATH); Saipal, 29°57'51"N, 81°13'6"E, 31 Jul. 1976, *H. Tabata, K.R. Rajbhandari & K. Tsuchiya* 4028 (KATH). **Bajura**: Chauki Lekh, 29°35'34"N, 81°38'5"E, 4427 m, 15 Aug. 2017, *BSH C42*; Chauki Lekh, 29°37'16"N, 81°34'30"E, 4427 m, 16 Aug. 2017, *BSH C52.***Dolakha**: Dudh Kunda, 4550 m, 5 Sep. 1983, *K.R. Rajbhandari 9915* (KATH). **Dolpa**, Nahure, 14,000 ft, 24 Jun. 1952, *Polunin, O.V., Sykes, W.R. & Williams, L.H.J. 1434*. **Jumla**: Maharigaon, 15,000 ft, 20 Jul. 1952, *Polunin, O.V., Sykes, W.R. & Williams, L.H.J. 226*. **Mustang**: Muktinath Himal, Muktinath Range, 28°44'37"N, 83°53'14"E, 480–5000 m, 18 Sep. 1981, *M.A. Farille 81-415* (E); Muktinath Himal, Muktinath Range, hab dans la praire alpine, 28°44'37"N, 83°53'14"E, 4800–5000 m, 18 Sep. 1981, *M.A. Farille 81-415* (E); Kaisang – Omang Kharka 3800 m, 1 Aug. 1996, *K.R. Rajbhandari 9672251* (KATH); Muktinath, 28°48'58"N, 83°51'47"E, 4180 m, 12 Oct. 1976, *H. Tabata, K.R. Rajbhandari, K. Tsuchiya & Y. Konno 6310* (KATH); Muktinath, alpine grassland, 28°48'58"N, 83°51'47"E, 4120 m, 12 Oct. 1976, *H. Tabata, K.R. Rajbhandari, K. Tsuchiya & Y. Konno 6342* (KATH). **Myagdi**: 28°32'0"N, 83°13'0"E, 3360 m, 20 Sep. 1996, *M. Mikage, R. Hirano, A. Takahashi & K. Yonekura 9682900* (KATH). **Ramechhap**: Thare Og, 27°45'N, 86°28'E 24 Jul. 1985, *H. Ohba, M. Wakabayashi, M. Suzuki, N. Kurosaki, K.R. Rajbhandari & S.K. Wu 60657* (KATH). **Rasuwa**: Gosainkund, 28°4'58"N, 85°24'51"E, 4300 m, 27 Jul. 1995, *T. Hoshino, K. Dan, H. Koba, Y. Omori, C.P. Rauniyar, M. Sato, P. Shrestha & S. Takatsuki 9537190* (KATH); Gosainkund, 4350 m, 28°4'58"N, 85°24'51"E, 27 Jul. 1995, *T. Hoshino, K. Dan, H. Koba, Y. Omori, C.P. Rauniyar, M. Sato, P. Shrestha & S. Takatsuki 9537218* (KATH); Gosainkund, 28°4'58"N, 85°24'51"E, 4300 m, 28 Jul. 1995, *T. Hoshino, K. Dan, H. Koba, Y. Omori, C.P. Rauniyar, M. Sato, P. Shrestha & S. Takatsuki 9537221*, *9537*–*222* (KATH). **Rukum**: Chalikhe Pahar, near Chalike Pahar, exposed south facing slopes, 28°40'N, 83°4'E, 4240 m, 17 Jun 1954, *J.D.A. Stainton, W.R. Sykes & L.H.J. Williams 3163* (E). **Solukhumbu**: Seto Pokhari (4810m) – Chhomalang Base Camp (4495), 27°47'N, 86°57'E, 4810 m, 17 Aug. 1995, *F. Miyamoto, M. Amano, H. Ikeda, C.M. Joshi, K. Arai & T. Komatsu 9592313* (E); Beni, alpine meadow, 27°32'59"N, 86°35'13"E 4600 m, *K. Tsuchiya 42693* (KATH).

#### Ptilagrostis yadongensis

Taxon classificationPlantaePoalesPoaceae

Keng & Tang, J. SouthW. Agric. Univ. 4: 44 (1985).

B5B8AF8B78065399A4AAFEED612B452E

≡ Ptilagrostis macrospicula Cai, Acta Bot. Boreal.-Occid. Sin. 23(11): 2018 (2003). superfl. name.
= Stipa milleri Noltie, Edinburgh J. Bot. 56(2): 288 (1999); ≡ Ptilagrostis milleri (Noltie) M.Nobis & A.Nobis, Nordic J. Bot. 31: 623 (2013).

##### Type.

China. Xizang: Yadong, 14 Sep. 1974, *Qinghai-Xizang Exped. 74–2496* (lapsus calami as *74–2469*; holotype HNWP, isotype PE).

##### General distribution.

Bhutan, China (Tibet), Nepal (Noltie 1999; Nobis and Nobis 2013; Nobis et al. 2015; Zhang et al. 2016).

##### Distribution in Nepal.

Bajhang, Dolkha, Rasuwa, Solukhumbu.

##### Habitat.

Alpine meadows, moist grassy places, under shrubs, swampy places, *Kobresia* moors.

##### Altitudinal range.

3600–4600 m.

##### Note.

These specimens were previously identified as *P. concinna* which also occurs in Nepal and which can be distinguished by its subequal glumes, lemma and palea. It has also been confused with *Ptilagrostis bhutanica* (Noltie) M.Nobis (basionym: *Stipa bhutanica*Noltie 1999: 289; Nobis et al. 2016), from Bhutan and China. However, these two taxa differ in the upper part of the awn which is scabrous in *P. bhutanica* and shortly pilose (with hairs over 0.5 mm long) in *P. yadongensis*.

##### Selected specimens studied.

**Bajhang**: Saipal Aletsoura, 29°57'51"N, 81°13'6"E, 4333 m, 31 Jul. 1976, *H. Tabata, K.R. Rajbhandari & K. Tsuchiya 1941* (KATH). **Dolkha**: Rolwaling Dudh Kunda, 4520 m, 15 Jul. 1975, *P.R. Shakya, K.R. Rajbhandari & H.K. Saiju 75/2978* (KATH). **Rasuwa**: Ya La, 3600 m, 29 July 1972, *A.Maire, AMA 250* (E); Upper Langtang, 4600 m, 30 Sep. 1986, G. Miehe 13090 (KATH). **Solukhumbu**, Chola Tsho, north side of lake, SE facing slope, rocks and sand near lakeside, Juniperus indica dwarf scrubland with Rhododedron setosum and Potentilla fruticosa, 27°55'18"N, 86°47'50"E, 4500 m, 21 Sep. 2005, *DNEP3 AX98* (E, KATH).

#### Ptilagrostis concinna

Taxon classificationPlantaePoalesPoaceae

(Hook. f.) Roshev., Fl. URSS 2: 75 (1934).

D4DFB9F5CE2D5513A17F0D15FEF3A942

Stipa concinna Hook. f., Fl. Brit. India 7(22): 230 (1897) (Basionym).

##### Type.

Sikkim-Himalaya, Tibetan region, 14000–16000ft, 1861, *Hooker* (holotype K!, isotypes G, GOET!, LE 9267!).

##### General distribution.

Himalayas: China (Tibet), India (Ladakh and Sikkim), Nepal (Freitag 1985; Wu and Phillips 2006).

##### Distribution in Nepal.

Solukhumbu, Mustang.

##### Habitat.

Alpine meadows, moist grassy places, under shrubs, swampy places, *Kobresia* moors.

##### Altitudinal range.

4400–5300 m.

##### Selected specimens studied.

**Solukhumbu**: Seto Pokhari, 27°47'N, 86°55'E, 4495–4810 m, 17 Aug. 1995, *F. Miyamoto, M. Amano, H. Ikeda, C.M. Joshi, K. Arai & T. Komatsu 9592313* (KATH). **Mustang**: Thorung La, 5200–5300 m, 19 Sep. 1981, *M.A. Farille 81-434* (E 189114).

#### Achnatherum

Taxon classificationPlantaePoalesPoaceae

P.Beauv., Ess. Agrostogr.: 19, 146, pl. 6, f. 7 (1812)

85EF46530AB25B3DA65E13306CD5B935

##### Type.

*Achnatherum calamagrostis* (L.) P.Beauv.

### Key to the genus *Achnatherum*

**Table:** 

1	Glumes distinctly unequal. Lemma apex with ring of hairs over 3 mm long. Callus 0.5–0.7 mm long, acute at the apex	***A. staintonii***
–	Glumes equal or almost so. Lemma apex with ring of hairs up to 2 mm long. Callus up to 0.5 mm long, rounded at the apex	**2**
2	Lemma and palea clearly unequal. Leaves filiform, inrolled. Culms up to 45 cm long. Panicle with very short branches	***A. jaquemontii***
–	Lemma and palea subequal. Leaves flat. Culms over 60 cm long. Panicle with widely spreading branches	***A. brandisii***

#### Achnatherum brandisii

Taxon classificationPlantaePoalesPoaceae

(Mez) Z.L.Wu, Acta Phytotax. Sin. 34: 154 (1996).

3D9DDBA3171558078E48A5CF71B2C084

Stipa brandisii Mez, Repert. Spec. Nov. Regni Veg. 17(13–18): 207 (1921) (Basionym).
= Stipa subeffusa Ohwi, Acta Phytotax. Geobot. 17: 15 (1957).

##### Type.

[India] N. W. Himalaya, Kulla, Oct. 1876, *Brandis* 1005 (lectotype K 32092! selected and labeled by H. Freitag on 15 March 1984 but **designated here**).

##### General distribution.

Afghanistan, Bhutan, China, NW India, Nepal, Pakistan (Freitag 1985; Wu and Phillips 2006).

##### Distribution in Nepal.

Dolpa, Manang, Mustang.

##### Habitat.

Open dry slopes, among shrubs and in Bamboo (*Sinarundinaria* sp.) thicket.

##### Altitudinal range.

2400–4000 m.

##### Note.

*Stipa brandisii* was described by Mez (1921) based on a specimen housed at B but destroyed during the Second World War. In his original description of the species Mez (1921) reported that the species had been collected in ‘Western-Himalaya, Kulla ([by] Brandis)’, but did not provide further information about the date of specimen collection, number and place where it was housed. In the absence of the original material, the specimen at K (http://apps.kew.org/herbcat/getImage.do?imageBarcode=K000032092) was selected as lectotype by H. Freitag in 1984 and subsequently cited as the holotype (Freitag 1985). We designate this here as the lectotype.

##### Selected specimens studied.

**Dolpa**: Above Chong, near Tibrikot Growing, among shrubs on open slopes, 29°1'40"N, 82°46'22"E, 2580 m, 11 Sep. 1952, *O.V. Polunin, W.R. Sykes & L.H.J. Williams 3314* (E). **Manang**: Humde, Bhraka, open place, 3400 m, Aug. 1983, *K.R. Rajbhandari 8786* (KATH); Boraga, Entre Braga et Ghyaru, 28°39'24"N, 84°2'22"E, 3500 m, 22 Sep. 1981, *M.A. Farille 81-486* (E); Boraga, Entre Braga et Ghyaru, bois ouvert xerophile, 28°39'24"N, 84°2'22"E 3500 m, 22 Sep. 1981, *M.A. Farille 81-491* (E); Boraga Annapurna Himal, Manang, Annapurna III, north-slope above Braga, on pastures, 28°39'24"N, 84°2'22"E, 3850 m, 14 Oct. 1969, *T. Wraber, 36404(502)* (BM). **Mustang**: Versant de Muktinath Range, faisant face a Jomsom, 50 m au dessus du village Rochers [on rocks], environnement rocheux, mais dans les touffes d’epineux oub d’Artemisia, 28°46'51"N, 83°43'27"E, 2750 m, 16 Sep. 1981, *M.A. Farille 81-358* (E); Ommang 28°44'N, 83°45'E, 3600 m, 31 Jul. 1996, *T. Hoshino, M. Amano, H. Koba, N. Miyoshi, K.R. Rajbhandari, M. Sato, P. Shrestha & S. Takatsuki 9662100* (KATH); Jomsom, 28°46'51"N, 83°43'27"E, 3350 m, 31 Jul. 1996, *T. Hoshino, M. Amano, H. Koba, N. Miyoshi, K.R. Rajbhandari, M. Sato, P. Shrestha & S. Takatsuki 9670079* (KATH); Kali Gandaki, Tangdung-Khola, S-facing, wind-blown slope, 2490–2480 m, Aug. 1977, *G. Miehe* (BM); Cha Lungpa, NE-facing slope, alpine pastures 3940 m, 27 July 1977, *G. Miehe 351b* (BM); NW of Tukche, valley of Yamkin Khola, in Bamboo (Sinarundinaria sp.) thicket, 28°41'15"N, 83°37'35"E, 2840 m, 20 Sep. 1995, *M. Mikage & K. Yonekura 9552331* (KATH); Tukucha (Kali Gandaki), 3180 m, [28°42'33"N, 83°38'37"E], 21 Aug. 1954, *J.D.A. Stainton, W.R. Sykes & L.H.J. Williams 7363* (BM, E 619028); Tukucha, Kali Gandaki, amongst hillside shrubs, 28°42'33"N, 83°38'37"E, 3030 m, 12 Sep. 1954, *J.D.A. Stainton, W.R. Sykes & L.H.J. Williams 7813* (E); Chimgaon (N of Tukucha) Kali Gandaki, on dry slopes, 28°43'38"N, 83°40'45"E, 2880 m, 14 Sep. 1954, *J.D.A. Stainton, W.R. Sykes & L.H.J. Williams 9887* (BM).

#### Achnatherum jacquemontii

Taxon classificationPlantaePoalesPoaceae

(Jaub. & Spach) P.C.Kuo & S.L.Lu, Fl. Reipubl. Popularis Sin. 9(3): 323, pl. 80, f. 15–19 (1987)

4269492C9CB159AABEA0C2FCE8A68D94

Stipa jacquemontii Jaub. & Spach, Ill. Pl. Orient. 4: 60, pl. 339 (1851) (Basionym).
≡ Lasiagrostis jacquemontii (Jaubert & Spach) Munro ex Boiss., Fl. Orient. 5: 506 (1884);
≡ Lasiagrostis jacquemontii (Jaub. & Spach) Munro ex Aitch., J. Linn. Soc., Bot. 18: 107 (1880).
= Stipa jacquemontii Jaub. & Spach subsp. chuzomica Noltie, Edinburgh J. Bot. 56(2): 290, f. 1Q–U (1999).

##### Type.

[India] ad ruped in excelsis Emodi Cashemyrianim 2750 m, 1831, *Jacquemont 994*, (holotype P, isotype K!).

##### General distribution.

E Afghanistan, Bhutan, China (Tibet), NW India, Nepal, Pakistan (Freitag 1985; Noltie 2000; Wu and Phillips 2006).

##### Distribution in Nepal.

Mustang.

##### Habitat.

Dry mountain slopes, especially in rock crevices.

##### Altitudinal range.

2500–3000.

##### Selected specimens studied.

**Mustang**: Marpha, pentes rocheues arides, 28°45'11"N, 83°41'28"E, 2650 m, 16 Sep. 1981, *M. Farille 81-340* (E); Versant de Muktinath Range, faisant face a Jamson, 100 m au dessus du Village Rochers, 2800 m, 16 Sep. 1981, *M.A. Farille 81-347* (E 188712); Barsumg Khola, on a dry cliff, 28°52'N, 83°16'E, 10000 ft, 18 Jul. 1963, *J.D.A. Stainton 4417* (E 00619022).

#### Achnatherum staintonii

Taxon classificationPlantaePoalesPoaceae

(Bor) M.Nobis & P.D.Gudkova, comb. nov .

03E8B22273C3536CAF0093D84D0D9F5A

urn:lsid:ipni.org:names:77200950-1

Stipa staintonii Bor, Bull. Bot. Surv. India 7: 133 (1965) (Basionym).
≡ Stipella staintonii (Bor) Röser & Hamasha, Pl. Syst. Evol. 298: 365 (2012), nom. inval.;
≡ Stipellula staintonii (Bor) Röser & H.R. Hamasha, Schlechtendalia 24: 92 (2012).

##### Type.

Nepal, near Seng Khola, 12500 ft [3810 m.], exposed cliffs, 4 Okt 1954, *Stainton, Sykes & Williams 4677* (holotype K!, isotype BM!).

##### General distribution.

Nepal (endemic; Bor 1965; Freitag 1985).

##### Distribution in Nepal.

Baglung, Dolpa, Manang, Mustang, Rukum.

##### Habitat.

open rocky or stony sandy slopes and scrublands.

##### Altitude.

3000–4200 m.

##### Note.

Although *Achnatherum staintonii* has been confused with *Stipa przewalskyi*, the latter species does not occur in Nepal. *Achnatherum staintonii* is easily distinguished from *Stipa przewalskyi* by having maize-like vs. saw-like LEPs and in having unequal glumes and distinctly longer lemma than palea vs. glumes as well as lemma and palea subequal, respectively. Röser (2012) transferred five species of *Stipa*, including *Stipa staintonii*, into their new genus *Stipellula* on the basis of his earlier molecular analysis (Hamasha et al. 2012). *Stipellula* is characterized by its maize-like lemma epidermal pattern which clearly distinguishes it from *Stipa* and confirms that these species belong to the achnatheroid group of grasses within the Stipeae. However, there are no unique, diagnostic morphological characters to separate *Stipellula* from *Achnatherum* which is itself polymorphic and highly polyphyletic (Romaschenko et al. 2012; Hamasha et al. 2012). Thus, we prefer to treat *Stipa staintonii* as a member of *Achnatherum*.

##### Selected specimens studied.

**Baglung**: Sing Khola, wet rocky cliff, 18 Sep. 1976, *H. Tabata, K.R. Rajbhandari & K. Tsuchiya 3711* (KATH). **Dolpa**: Ringmo, dry hillslope, 29°10'20"N, 82°55'50"E, 3400 m, 2 Aug. 1973, *S. Einarsson, L. Skärby & B. Wetterhall 3128* (BM); Barbung Khola, 28°52'N, 83°15'E, 3030 m, 18 Jul. 1963, *J.D.A. Stainton 4417* (BM); Barbung Khola, 28°52'N, 83°18'E, 3030 m, 13 Jul. 1963, *J.D.A. Stainton 4417* (E); Suligad, Rhagaon, rocky slope, 29°28'N, 82°55'E, 2600 m, 25 Sep. 1982, *K.R. Rajbhandari & K.J. Malla 6740* (KATH); Karnali, Ringmigaon, on dry hilslope, 3400 m, 2 Aug. 1973, *S. Einarsson, L. Skärby, B. Wetterhall 3126* (UPS); Karnali, **Manang**: Bhraka, Humde, open rocky slope, 28°38'24"N, 84°5'36"E, 3400 m, 3 Aug. 1983, *K.R. Rajbhandari 8814* (KATH); Marsyandi valley, Tangi above Manangbhot, on stony sandy places, 28°39'56"N, 84°1'33"E, 3800 m, 12 Oct. 1969, *T. Wraber 36427* (E); Tangi, above Manangbhot, Tangje, on stony sandy places, 28°39'22"N, 84°2'2"E, 3800 m, 12 Oct. 1969, *T. Wraber 479* (BM). **Mustang**: Entre Marpha et Syang; Syang, Marpha, 28°45'11"N, 83°41'28"E, 2650 m, 16 Sep. 1981, *M.A. Farille 81-340* (E); Entre Larjung et Tukuche, 2550 m, 14 Sep. 1981, *M.A. Farille 81-313* (E); Cha Lungpa, in E-facing Cupressus forest, 3030 m, 3 Oct. 1977, *G. Miehe 80* (BM); Phalyak, dry place 28°49'24"N, 83°44'23"E, 4110 m, 9 Aug. 2002, *F. Miyamoto, N. Kurosaki, S. Akiyama, H. Ikeda, Y. Iokawa, Y. Takahashi, M. Tsusaka & M.N. Subedi 20210022* (KATH); Phalyak to Pongio Kharka, 28°49'0"N, 83°45'0"E, 3800–4000 m, 9 Aug. 2002, *F. Miyamoto, N. Kurosaki, S. Akiyama, H. Ikeda, Y. Iokawa, Y. Takahashi, M. Tsusaka & M.N. Subedi 20220034* (KATH); Chele – Samar, 28°57'43"N, 83°48'6"E, 3450-3670 m, 4 Aug. 2001, *S. Noshiro, M. Amano, Y. Iokawa, T. Kurosawa, & M.N. Subedi 20105005* (KATH); Tukuche, open rocky slope, 28°42'33"N, 83°38'37"E, 2630 m, 19 Jul. 1983, *K.R. Rajbhandari 7991* (KATH); Jomsom, open slope, 28°46'51"N, 83°43'27"E, 2760 m, 21 Jul. 1983, *K.R. Rajbhandari 8072* (KATH); Jomsom to Sayang, cliffs in dry valley with scrub (Caragana, Clematis, Rosa etc.), 28°46'51"N, 83°43'27"E, 2700 m, 19 Sep. 1999, *Shrestha et al. 1014* (E); Tukuche, 28°42'33"N, 83°38'37"E, 3180 m, 21 Aug. 1954, *J.D.A. Stainton, W.R. Sykes & L.H.J. Williams 7352* (BM). **Rukum**: Sen Khola, 28°42'54"N, 82°57'21"E, 3790 m, 4 Oct. 1954, *J.D.A. Stainton, W.R. Sykes & L.H.J. Williams 4677* (BM).

#### Piptatherum

Taxon classificationPlantaePoalesPoaceae

P.Beauv., Ess. Agrostogr.: 17: 173 (1812)

8D508D06821A50ABB32678E3FF54DED1

##### Type.

*Piptatherum coerulescens* (Desf) P. Beauv.

### Key to the genus *Piptatherum*

**Table:** 

1	Panicle compressed	**2**
–	Panicle lax	**3**
2	Awn terminal	***P. laterale***
–	Awn subterminal	***P. gracile***
3	Lemma almost equal to glumes, apical part gradually narrowed into a persistent awn	***P. aequiglume***
–	Lemma much shorter than glumes, apical part abruptly contracted into a slender, caducous awn	***P. munroi***

#### Piptatherum aequiglume

Taxon classificationPlantaePoalesPoaceae

(Duthie ex Hook.f.) Roshev., Bot. Mater. Gerb. Bot. Inst. Komarova Akad. Nauk SSSR 14: 113 (1951).

4C8C8A4D319A5CDF8CDD4F224F30C1CD

Oryzopsis aequiglumis Duthie ex Hook. f., Fl. Brit. India 7(22): 234 (1896) (Basionym).
= Piptatherum sinense Mez, Repert. Spec. Nov. Regni Veg. 17(486–491): 211 (1921).

##### Type.

India: distr. Jansar, Gamble 15143 (lectotype K! designated by Bor 1970).

##### General distribution.

Afghanistan, Bhutan, S China, NW India, Nepal, Pakistan (Freitag 1975; Noltie 2000; Wu and Phillips 2006).

##### Distribution in Nepal.

Rukum.

##### Habitat.

Moist mesophytic forests.

##### Altitude range.

3500–4000 m.

##### Selected specimens studied.

**Rukum**: near Dogadi Khola, 3660 m, 8 Aug. 1954, *J.D.A. Stainton, W.R. Sykes & L.H.J. Williams 3801* (E 814753); nr. Dogadi Khola, 3790 m, 8 Aug. 1954, *J.D.A. Stainton, W.R. Sykes & L.H.J. Williams 3794* (E 814768).

#### Piptatherum gracile

Taxon classificationPlantaePoalesPoaceae

Mez, Repert. Spec. Nov. Regni Veg. 17(486–491): 211 (1921).

B02F431AD12A5D1E98D6AEEFF372846A

≡ Oryzopsis gracilis (Mez) Pilg., Notizbl. Bot. Gart. Berlin-Dahlem 14: 347 (1939:).

##### Type.

Tibet occ., 3900–4000 m., *Thomson* s.n. (lectotype W designated by Freitag 1975, isolectotype K!).

##### General distribution.

Afghanistan, China, N India, Nepal, Pakistan, Tajikistan (Freitag 1975; Wu and Phillips 2006).

##### Distribution in Nepal.

Mustang.

##### Habitat.

alpine steppes and meadows.

##### Altitude range.

2500–4000 m.

##### Selected specimens studied.

**Mustang**: Marpha, 28°45'11"N, 83°41'28"E, 2670 m, 16 Sep. 1981, *M.A. Farille 81-336* (E); Jomsom, 28°46'51"N, 83°43'27"E, 3200 m, Versant de Muksant Range, faisant face a Jomsom, 500 m au dessus du village, on rocks, 16 Sep. 1981, *M.A. Farille 81-352* (E); Jharkot – Kagbeni, 28°50'17"N, 83°47'3"E, 2800–3550 m, 17 Sep. 1981, *M.A. Farille 81-359* (E); 10 Jul. 2000, *Y. Iokawa, M.N. Subedi, Y. Takahashi & K. Kano 20020054* (E); Dzong Pura (Muktinath), 28°49'41"N, 83°51'19"E 3640 m, 29 July 1954, *J.D.A. Stainton, W.R. Sykes & L.H.J. Williams 2087* (E); Tange, 29°0'38"N, 83°56'45"E, 3640 m, 1 Aug. 1954, *J.D.A. Stainton, W.R. Sykes & L.H.J. Williams 2125* (E); Kagbeni, 28°50'17"N, 83°47'3"E, 3030 m, 8 Jun 1954, *J.D.A. Stainton, W.R. Sykes & L.H.J. Williams 5659* (E); Ekle Bhatti, on sunny rocky steep slope at pathside, 2270 m, 22 Sep. 1995, *M. Mikage et al. 9552384* (E 224287).

#### Piptatherum munroi

Taxon classificationPlantaePoalesPoaceae

(Stapf ex Hook.f.) Mez, Repert. Spec. Nov. Regni Veg. 17: 212 (1921).

43B27AB79232504B84252E23FD0F4059

Oryzopsis munroi Stapf ex Hooker, Fl. Brit. India 7(22): 234 (1897) (Basionym).
= Oryzopsis stewartiana Bor, Kew Bull., 272 (1953);
= Oryzopsis geminiramula Ohwi, Acta Phytotax. Geobot. 17: 14 (1957).

##### Type.

NW India, Chenab Himalayas, 1852, Thomson (lectotype E 360583!, designated by Freitag 1975).

##### General distribution.

China, N India, Kashmir, Nepal (Freitag 1975).

##### Distribution in Nepal.

Dolpa, Jumla, Mustang, Sankhuwasabha, Solukhumbu.

##### Habitat.

Among dwarf *Rhododendron* shrubland and in coniferous forest.

##### Altitude range.

3490–4500.

##### Selected specimens studied.

**Dolpa**: Sangdan, 28°55'N, 83°41'E, 4550 m, 21 Jul. 1963, *J.D.A. Stainton 4443* (E). **Jumla**: Maharigaon, grassy slope, 29°19'50"N, 82°22'15"E, 4090 m, 18 Jul. 1952, *O.V. Polunin, W.R. Sykes, & L.H.J. Williams 219* (E). **Mustang**: 2800 m, 16 Sep. 1981, *M. Farille, 81-348* (E); Lo Tsho Dhyum, Nr. Kali Grandaki River, Dhi (Dhee) area, stony river bank, scattered open vegetation, 22 Jul. 1998, *W.R. Sykes 285/98* (E). **Sankhuwasabha**: Thudam, 27°45'31"N, 87°32'59"E, 3490–3480 m, 2 Nov 1971, *L.W. Beer, C.R. Lancaster & D. Morris 10679* (E). **Solukhumbu**: Namche Bazar, along the trail to Phurte, South east facing grassy slopes, open grassy slopes with bushes of Juniperus, 27°48'24"N, 86°42'46"E, 3420 m, 13 Sep. 2005, *F.M. Watson et al. DNEP3 AX33* (E, KATH).

#### Piptatherum laterale

Taxon classificationPlantaePoalesPoaceae

(Regel) Munro ex Nevski, Trudy Bot. Inst. Akad. Nauk SSSR Ser. 1, Fl. Sist. Vyssh. Rast. 4: 217 (1937).

7A6543F74EDF50A7852EC47F8B0F0E78

Milium laterale Regel, Trudy Imp. S.-Peterburgsk. Bot. Sada 7: 645 (1881) (Basionym).
≡ Oryzopsis lateralis (Regel) Stapf ex Hook. f., Fl. Brit. India 7(22): 234 (1896);
≡ Piptatherum laterale (Regel) Roshev., Bot. Mater. Gerb. Inst. Bot. Akad. Nauk Kazahsk. SSR 14: 117 (1951).
= Oryzopsis pubiflora Hack., Denkschr. Kaiserl. Akad. Wiss., Wien Math.-Naturwiss. Kl. 50(2): 8 (1885); ≡ Piptatherum pubiflorum (Hack.) Roshev., Bot. Mater. Gerb. Bot. Inst. Komarova Akad. Nauk SSSR 14: 111 (1951).
= Oryzopsis vavilovii Roshev., Trudy Prikl. Bot. Selekts. 19(1): 123 (1928); ≡ Piptatherum vavilovii (Roshev.) Roshev., Bot. Mater. Gerb. Inst. Bot. Akad. Nauk Kazahsk. SSR 14: 118 (1951).

##### Type.

Afghanistan, Kurram valley, Sikarm, common at 3650 m., dry localities, 1879, *Aitchison* (holotype LE!, isotype K).

##### General distribution.

widely distributed species, occurring from Turkey up to Bhutan and S China (Freitag 1975, Tzvelev 1976).

##### Distribution in Nepal.

Although we did not find any specimens of *Piptatherum laterale* during this study, it is known from nearby regions of Bhutan, China, India (Freitag 1975, Noltie 2000, Wu and Phillips 2006), so it is very likely also to be present in Nepal.

#### Trikeraia

Taxon classificationPlantaePoalesPoaceae

Bor, Kew Bull. 9(4): 555, f. s.n. (1954)

26EA1D242497518581DD29657E3DE724

##### Type.

*Trikeraia hookeri* (Stapf) Bor.

#### Trikeraia hookeri

Taxon classificationPlantaePoalesPoaceae

(Stapf) Bor, Kew Bull. 9(4): 555–556 (1954).

72673E9169B852D2BBED1C13FC8CB9CD

Stipa hookeri Stapf, J. Linn. Soc., Bot. 30: 120 (1894) (Basionym).
≡ Achnatherum hookeri (Stapf) Keng, Claves Gen. Sp. Gram. Prim. Sinic. 106, 213 (1957).
= Timouria aurita Hitchc., J. Wash. Acad. Sci. 23: 134 (1933).

##### Type.

Tibet, 4500 m, sheltered nullahs near water, Jul-Sep. 1891, Thorold 124 (holotype K!, isotype C).

##### General distribution.

China (Tibet), India (Sikkim, Ladakh), Pakistan (Freitag 1985; Wu and Phillips 2006).

##### Distribution in Nepal.

Although we did not find any specimens of *Trikeraia hookeri* during this study we include it here because it is known from nearby regions (Freitag 1975, Wu and Phillips 2006), so it is very likely to be present in Nepal. Freitag (1985) reported this species from Nepal based on *Sufed 104* (K), Mt. Everest, Tinkye palin, 4270 m, but this specimen appears to have been collected on the Tibetan side of Mt Everest.

##### Habitat.

Scrublands, alpine mats.

##### Altitude range.

4000–4300 m.

##### Note.

The 2005 DNEP3 expedition to Solukhumbu collected several specimens which were identified as *Trikeraia oreophila* Cope by H. Noltie [Dingboche, on trail south to the Lobuche Khola bridge, east facing valley side, 27°52'50"N, 86°49'7"E, 4230 m, 23 Sep. 2005, *M.F. Watson et al. DNEP3 AX107* (E, KATH)]. These specimens are characterized by having 4–6 mm long lemma lobes, the awn arising below the middle of the lemma and ovary with two stigmas. Recently, *Trikeraia oreophila* was found to be conspecific with *Sinochasea trigyna* Keng (WCSP 2019). Morphological and molecular studies have shown that the genus *Sinochasea* is distinct not only from *Trikeraia*, but also from all the other genera of the tribe Stipeae, and therefore it was transferred to the tribe *Phaenospermateae* Renvoize & Clayton (Schneider et al. 2011; Romaschenko et al. 2012; Kellogg 2015).

## Supplementary Material

XML Treatment for Orthoraphium

XML Treatment for Orthoraphium roylei

XML Treatment for Stipa

XML Treatment for Stipa breviflora

XML Treatment for Stipa klimesii

XML Treatment for Stipa krylovii

XML Treatment for Stipa capillacea

XML Treatment for Ptilagrostis

XML Treatment for Ptilagrostis duthiei

XML Treatment for Ptilagrostis dichotoma

XML Treatment for Ptilagrostis yadongensis

XML Treatment for Ptilagrostis concinna

XML Treatment for Achnatherum

XML Treatment for Achnatherum brandisii

XML Treatment for Achnatherum jacquemontii

XML Treatment for Achnatherum staintonii

XML Treatment for Piptatherum

XML Treatment for Piptatherum aequiglume

XML Treatment for Piptatherum gracile

XML Treatment for Piptatherum munroi

XML Treatment for Piptatherum laterale

XML Treatment for Trikeraia

XML Treatment for Trikeraia hookeri
